# Meta-analysis protocol on the effects of cover crops on pool specific soil organic carbon

**DOI:** 10.1016/j.mex.2023.102411

**Published:** 2023-10-02

**Authors:** Julia Fohrafellner, Sophie Zechmeister-Boltenstern, Rajasekaran Murugan, Katharina Keiblinger, Heide Spiegel, Elena Valkama

**Affiliations:** a BIOS Science Austria, Silbergasse 30, Vienna 1190, Austria; b Department of Forest- and Soil Sciences, Institute of Soil Research, University of Natural Resources and Life Sciences Vienna, Peter Jordan Straße 82, Vienna 1190, Austria; c Department for Soil Health and Plant Nutrition, Institute for Sustainable Plant Production, Austrian Agency for Health and Food Safety, Spargelfeldstraße 191, Vienna 1220, Austria; d Natural Resources Institute Finland (Luke), Bioeconomy and Environment, Sustainability Science and Indicators, Tietotie 4, Jokioinen 31600, Finland

**Keywords:** EJPSOIL, Effect size, MAOC, MBC, POC, SOC, Synthesis, Meta-analysis protocol

## Abstract

Soil organic carbon (SOC) plays an important role in agricultural soils, as it contributes to overall soil health as well as climate change mitigation and adaptation. By conducting a meta-analysis, we aim to quantitatively summarize research studying the effects of cover crops (CC) on SOC pools throughout soil depths in arable cropland. We included global studies located in the climatic zones present in Europe. The pools chosen for this analysis are the particulate organic carbon (POC) and the mineral associated organic carbon (MAOC) and the microbial biomass carbon (MBC). Alongside, we will study the effects of a broad range of moderators, such as pedo-climatic factors, other agricultural management practices and CC characteristics e.g., type.

We identified 71 relevant studies from 61 articles, of which mean values for SOC pools, standard deviations and sample sizes for treatments (CC) and controls (no CC) were extracted. To perform the meta-analysis, an effect size will be calculated for each study, which will then be summarized across studies by using weighing procedure. Consequently, this meta-analysis will provide valuable information on the state of knowledge on SOC pool change influenced by CC, corresponding quantitative summary results and the sources of heterogeneity influencing these results.

## Review Highlights

• This is a protocol for a high-quality meta-analysis, studying the effects of cover crops on the POC, MAOC and MBC pools.
• We describe the complete process from the identification of the topic to the statistics which are going to be used to conduct the meta-analysis.
• By publishing this protocol in a peer-reviewed journal, we aim to make our research plans openly available and discussable, thereby raising the standards for conducting meta-analyses in soil and agricultural research.

## Graphical abstract

**Figure:**
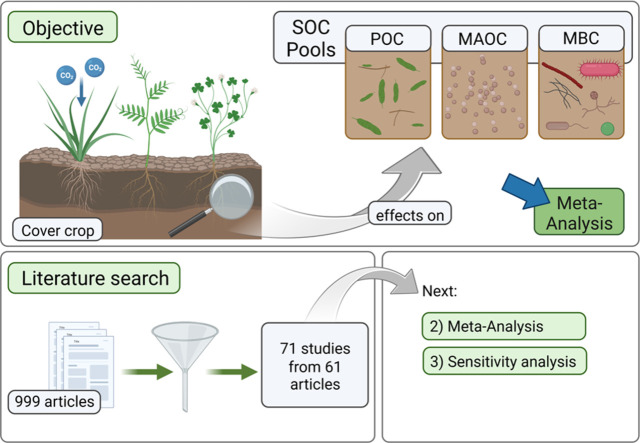

Specifications table

**Table:** 

Subject area:	Agricultural and Biological Sciences Please Select Subject Area from dropdown list
More specific subject area:	Soil organic carbon
Name of the reviewed methodology:	Meta-analysis
Keywords:	Effect size; MAOC; MBC; POC; SOC; synthesis
Resource availability:	This protocol was created according to the checklist of PRISMA-P [1] and PRISMA-EcoEvo [2].
Review question:	N/A

## Identification of the topic

Cover cropping is an alternative to leaving agriculturally managed soils bare, especially during the winter time. In case cover crops (CC) are winter-hardy, termination by e.g., tillage or pesticide application are viable options before sowing the following main crop [3], [4], [5]. Amongst other beneficial aspects, such as reducing soil erosion, increasing biodiversity [6], reducing N losses [7] or improving overall soil quality [8], it is evident that CC have a positive impact on soil organic carbon (SOC) [9], [10], [11]. Therefore, they are an effective measure to increase SOC contents in agricultural soils [5,12]*.* There are already numerous meta-analyses that quantitively synthesized the effects of CCs on total SOC globally [5,[12], [13], [14], [15], [16]] and in the Mediterranean climate [17].

What is not well understood so far is how stable the carbon is stored under CC cultivation. As total SOC is not the most sensitive indicator to describe changes in SOC stocks [18], it is failing to explain whether carbon is stored long- or short-term. More suited to give insight into these C dynamics are the particulate organic carbon (POC) and the mineral-associated organic carbon (MAOC) pool. POC largely consists of lightweight, undecomposed fragments whereas MAOC is built up by single molecules or microscopic fragments of organic material. They differ also between their mean residence time, which can range from years to decades for POC and decades to centuries for MAOC [19]. Both pools are more sensitive to changes and provide a deeper insight into the persistence of SOC compared to total SOC [19,20]. Another C fraction, which is tightly connected to the MAOC, is the microbial biomass carbon pool (MBC), as microbes and extracellular enzymes can attach to mineral surfaces which simultaneously facilitate bacterial growth [21,22]. Together the POC, MAOC and MBC pool contribute to an improved understanding of the fate of organic carbon sequestered by CC.

So far, only few meta-analyses studying CC effects on SOC on a pool level are available and, in some cases, their results are contradictory. First, McDaniel et al. [23] found in their quantitative synthesis that neither the inclusion of CCs nor moderators, as amount of N fertilizer or number of legumes in the rotation, had a significant impact on MBC. Contrary, Ma et al. [24] observed a positive impact of CC on MBC in their synthesis, but missed to analyse moderator effects. Similarly, Muhammad et al. [25] found in their meta-analysis a positive effect of CC on MBC, but did extract many observations per study, causing non-independent effect sizes. Regarding the POC and MAOC pool, there are very recent global meta-analyses published [26,27]. Both of these studies found positive effects of cover crops on the MAOC, POC and MBC pools. Nevertheless, these studies did not extract studies independent from each other and estimated standard deviation.

We aim to produce the first global meta-analysis that only includes studies conducted in climate zones relevant to Europe, which is studying the effects on CC on the MAOC, POC and MBC pool. Meta-analytical quality criteria will be followed [28], [29], [30], [31], including independent study extraction and computation of standard deviations with the EX-TRACT tool [32]. By doing so, we want to provide high quality and novel insights into CC effects on SOC pools tailored to European conditions.

## Objective

The objective of this protocol is to describe the methodology used for conducting this meta-analysis, studying the effects of CC on selected SOC pools in cropland soils. The following describes the formulated **research questions** of the meta-analysis:

1. How do CC affect particulate organic carbon (POC), mineral associated organic carbon (MAOC) and microbial biomass carbon (MBC) of cropland soils?
2. How do CC influence pool specific SOC throughout the soil profile (down to 100 cm)?
3. How do CC characteristics (type, species number, termination time, etc.) affect pool specific SOC?
4. How do agricultural management practices (soil tillage, fertilizer types and amounts, irrigation, etc.) affect pool specific SOC in presence of CC?
5. How do pedo-climatic factors (clay content, initial C content, annual average rainfall and temperature, etc.) affect pool specific SOC in presence of CC?

The research questions are structured according to the PICO framework (population, intervention, comparator, and outcome):

**Table:** 

*Population:*	Arable cropland, growing annual cereal crops, located in the climatic zones present in Europe according to Köppen-Geiger climatic zones as described in Kottek et al. [33]
*Intervention*:	Cover crop(s)
*Comparator*:	No cover crop(s) (e.g., bare fallow or main crop residues)
*Outcome*:	Pool specific SOC contents up to 1 m soil depth

Initially, we were planning to focus the meta-analysis on experiments conducted in Europe. As not enough literature on this topic is available for European experiments, we decided to expand our search to a global level. Only experimental sites located in climatic zones by Kottek et al. [33], which are also present in Europe, will be considered. These are BSh, BSk, BWh, Cfa, Cfb, Cfc, Csa, Csb, Csc, Dfa, Dfb, Dfc. Similarly, available data on the MAOC and POC pool was scarce, which lead to the inclusion of organic matter data, namely MAOM and POM. As described later in Section 6. “Data extraction and synthesis”, we will use log response ratio for effect size calculation, which allows to summarize values with a large variation across studies [31]. Therefore, effect sizes, calculated from organic matter or organic carbon, can be compared with each other. We further will conduct a subgroup analysis, studying whether data provision in form of organic matter or carbon is impacting our overall results (see Table 3 “Explanatory variables (moderators) and their ranges or groups”). To enhance the readability of this paper, we will simplify our terminology by referring to both organic carbon and matter (MAOC, MAOM and POC, POM) as “MAOC” and “POC”.

## Literature search strategy and data management

The search string was adapted after Haddaway et al. [34], who studied the effects of management practices on SOC in boreo-temperate systems. Web of Science core collection, Bielefeld Academic Search engine (BASE), Scopus, MDPI and ScienceDirect were searched for relevant scientific literature in April of 2022. Grey literature, which is literature not published by commercial publishers, but by institutions where publishing is not the primary activity [35], was searched for in Google Scholar and at Biorxiv.org. The mentioned databases used for identified in the papers by Gusenbauer and Haddaway [36] and Haddaway and Bayliss [37]. The search was conducted in English language only. The reference lists of published reviews on relevant topics were screened to check for additional articles which were missed by the search engines. In Table 1 the search string which was applied in Web of Science can be found. For the other searched databases, short versions of this search string were used, as the number of words possible to search are restricted.

**Table 1:** Search string for literature research in Web of Science Core Collection. Adapted after Haddaway et al. [34].

Population terms	Boolean operator	Intervention terms	Boolean operator	Outcome terms	Boolean operator	Exclusion terms
soil* AND (agr* OR farm*)	AND	(diversif* OR (grass OR clover) ley* OR legume* OR intercrop* OR inter-crop* OR “intermediate crop*” OR cover-crop* OR catch-crop* OR “green manure” OR mixed-crop* OR undersown)	AND	(POM OR fPOM OR POC OR fPOC OR “particulate organic” OR MAOM OR MOM OR MAOC OR MinOM OR MASOC OR “mineral organic” OR “mineral-associated” OR oPOM OR “occluded POM” OR aggregate-occluded OR aggregate-associated OR microaggregate OR micro-aggregate OR “microbial biomass carbon” OR MBC)	NOT	(orchard OR forest OR fruit OR aquaculture OR aquiculture OR wood* OR vineyard OR arboricultu* OR horticult* OR olive OR *cane OR *tropic*)

On June 1st, 2023, the EJP SOIL Long-term field experiment database [38] was searched for additional suitable articles and two more studies were retrieved. Moreover, a second search round was conducted to access articles published since the initial literature search (April 2022) and June 2023. Lastly, search strings were adapted to find articles (a) studying upland rice and (b) using the terms “carbon in aggregates” and “fractions” as synonyms for carbon pools, as these were missing in the previous search strings.

All results from the search of scientific literature in the selected databases were transferred into the software JabRef 5.5, where duplicates were removed automatically. Additionally, a search for duplicates and removal of book chapters and faulty exports was conducted manually. Relevant grey literature from Google Scholar was identified online, as a download of the results of searches is not possible. For BioRxiv, grey literature was downloaded and screened for relevance offline. As both databases yielded a large number of results and a complete screening of all entries was not possible, they were sorted according to relevance and screened as long as entries were no longer showing any relevance to our subjective. Therefore, the first 100 and 30 entries were screened for relevance in Google Scholar and BioRxiv, respectively. After potentially relevant scientific and grey literature was identified, all studies were transferred into Microsoft Excel © (version 1808) and another automatic duplicate removal was conducted to guarantee no article was included more than once. Lastly, the results of this process were compared to the findings of other reviews to ensure that key literature was found and to include additional studies presented [26,^39^,40]. Finally, we assigned each study a unique ID to improve traceability throughout the screening processes. The complete documentation of the literature search strategy can be found in the Annex I.

## Screening and eligibility criteria

**Fig. 1:**
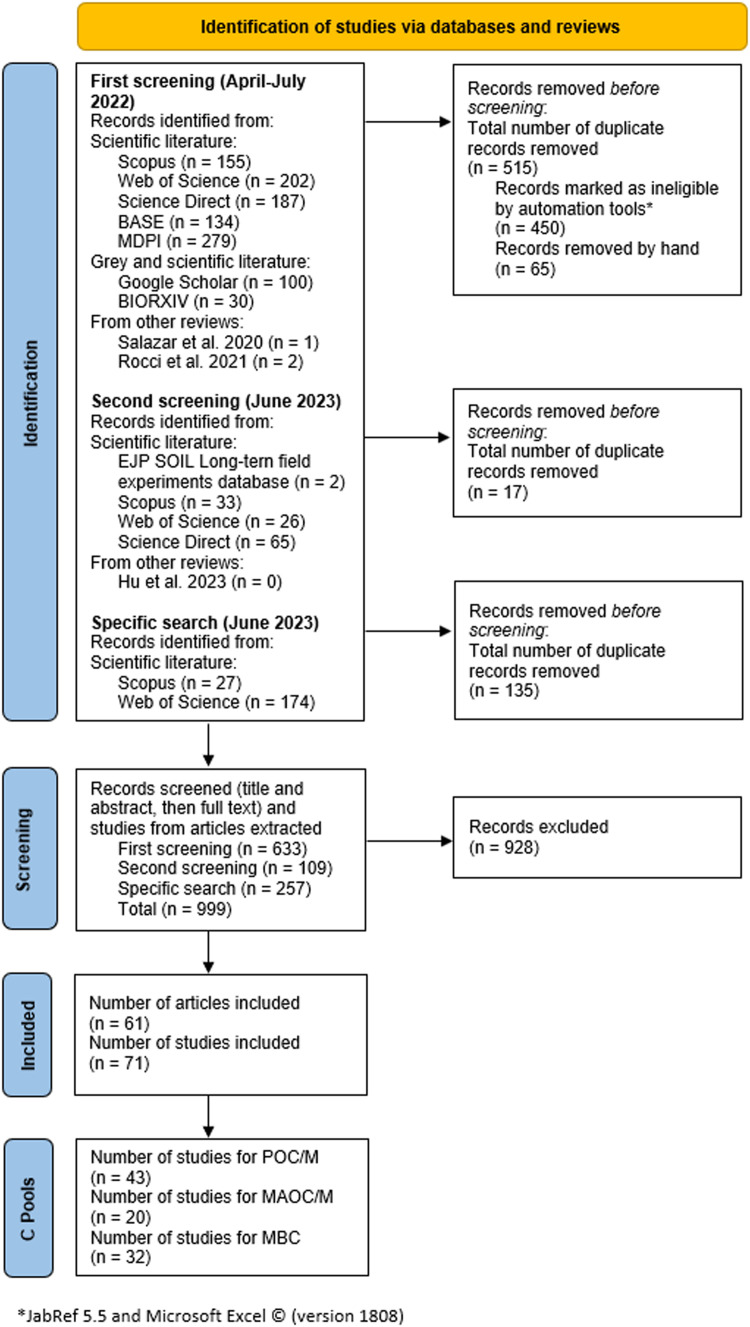
PRISMA Flow diagram of literature retrieval.

Each retrieved study was screened for relevance according to predefined inclusion and exclusion criteria (Table 2). These criteria are based on the PICO framework and formulated research questions and aim to ensure that included studies are comparable and therefore can be synthesized meta-analytically. When SOC results were presented as stock, we checked whether bulk density was measured or estimated and only the measured ones were included [31]. The screening was conducted in three steps. To check whether a study fit our scope, first, the title was examined for relevance. If it did not already indicate the presence of exclusion criteria, the abstract was screened. In a third stage, all studies which passed the abstract screening were checked for suitability (according to the eligibility criteria in Table 2) in form of a full text screening. Due to limitations in human resources, the screening process was conducted by the first author only. When decisions were unclear, the last author was asked for her opinion. The search for literature and the screening process is presented in form of a PRISMA flow diagram (Fig. 1).

**Table 2:** Eligibility criteria.

	Inclusion criteria (IC)	Exclusion criteria (EC)
1	English language	Other than English language
2	Control: No cover crop(s)	Control: Cover- or catch-crop(s) part of control; control is bare soil
3	Treatment: Cover crop(s)	Cover- or catch-crop(s) not part of treatment
4	Response variable: stock or concentration of MAOM/C, POM/C and MBC	Response variable other than MAOM/C, POM/C, MBC
5	Tillage in treatment and control the same	Tillage in treatment and control differ
6	Study period (of SOC measurement) one year or longer	Study period less than 1 year
7	Conducted on agricultural cropland with mineral soil (including: pure cereal crops; cereal + horticultural crops; upland [non-flooded] rice)	Other than agricultural cropland with organic soil (including: permanent grassland; grassland in rotation; pasture; pure horticulture; agroforestry; orchards; vineyards; flooded rice; cotton)
8	Field studies	Laboratory, mesocosm (e.g., greenhouse, litter bag) and modelling studies (unless primary data from field studies presented as well)
9	Climatic zone of globally conducted experiment is also present in Europe	Climatic zone of globally conducted experiment is not present in Europe
10	Bulk density was measured to calculate SOC stock	Bulk density was estimated or modelled to calculate SOC stock
11	Mean of treatment and control are available	Mean of treatment and control are not available
12	Standard deviation or standard error of treatment and control is stated or can be calculated with the tool by Acutis et al. [32]	Standard deviation is not stated and cannot be calculated with the tool by Acutis et al. [32]

## Effect moderators and sources of heterogeneity

To explain variation across studies, the effects of explanatory variables (moderators) on SOC pool response due to CC will be studied. The development of relevant moderators and their ranges or groups were based on a database provided by EJP Soil and adapted to our research scope. Selected moderators and their ranges or groups can be found in Table 3.

**Table 3:** Explanatory variables (moderators) and their ranges or groups.

Explanatory variable	Groups/ranges
**Agricultural management**	
Farming system	Conventional; organic
Tillage	Conventional tillage; reduced/minimum tillage; no-till
Cropping system	Monoculture; crop rotation
Number of main crop species in rotation	Continuous
Number of main crop species in treatment compared to control	Smaller; equal; higher
Presence of leguminous main crops in rotation	Yes; no
Crop rotation duration (years)	Continuous
Irrigation	Yes; no
Liming (t CaO ha^−1^ year^−1^)	Continuous
Inorganic N fertilizer	None; nitrogen; compound fertilizer
Inorganic N fertilizer rate (kg N ha^−1^ yr^−1^)	Continuous
Other inorganic fertilizer	None; phosphate; potassium; compound fertilizer; sulphur
Other inorganic fertilizer rates (kg ha^−1^ yr^−1^)	Continuous
Pesticides	Text
Herbicides	Text
Residue management of main crop	Left on field; incorporated; removed; partly removed; returned
Rate of residue incorporation of main crop (%)	Continuous
Organic matter (OM) input	None; green manure other than cover crop; livestock manure; biochar; slurry; compost; digestate; straw of main crop; guano; sludge; mix
Nitrogen input in form of OM (kg N ha^−1^ yr^−1^)	Continuous
Carbon input in form of OM (kg C ha^−1^ yr^−1^)	Continuous
**Cover crop characteristics**	
CC type(s)	Legumes; grasses; crucifers; others (composites, buckwheat family, waterleafs, linseeds); mixed
CC species number	Continuous
CC single grown or in mix	single; mixed
CC shoot-to-root ratio	Continuous
CC C/N ratio	Continuous
Frost resistance	Winter-hardy; freeze-killed; mixed
Seed rate (kg ha^−1^)	Continuous
Sowing time of CC (season)	Spring; summer; autumn; winter
CC peak biomass (Mg ha^−1^)	Continuous
CC average biomass (Mg ha^−1^)	Continuous
CC harvests per year	Continuous
Termination method	Herbicides; roller-crimper; ploughed; hand-hoeing; undercut; cultivator; grazed; shredded; none
CC harvest time (season)	Not harvested; spring; summer; autumn; winter
CC Termination time (season)	Spring; summer; autumn; winter
Years in rotation with CC	Continuous
Residue management of CC	Left on field; incorporated; harvested/removed; partly removed; returned
Main crops or fallow in control when CC in treatment	Fallow; type of main crop
**Experiment**	
Duration of experiment (years)	Continuous
SOC fractions measured in layer (cm)	Continuous
SOC fractions analytically measured by	Density; size; size and density; chloroform fumigation extraction; substrate induced respiration; microwave irradiation procedure; PLFA
MAOC size fraction (μm)	< 20; < 53
POC size fraction (μm)	50 – 2000 (total); 50 – 250 (micro); 250 – 2000 (macro)
MAOC density fraction (g cm^−3^)	> 1.6; > 1.85
POC density fraction (g cm^−3^)	< 1.05; < 1.6; <1.7; < 1.85
**Soil parameters at the beginning of experiment** Soil depth for measurement of soil parameters (cm)	0–100
Soil pH	Continuous
Soil texture class	Clay; loam; silt; sand 1
Clay (%)	Continuous
Silt (%)	Continuous
Sand (%)	Continuous
Clay content class	High >25 %; medium 15–25 %; low < 15 % 2
Initial SOC content (%)	Continuous
Bulk density (g cm^−3^)	Continuous
C/N ratio	Continuous
**Climate** Köppen-Geiger climatic zones in Europe	BSh; BSk; BWh; Cfa; Cfb; Cfc; Csa; Csb; Csc; Dfa; Dfb; Dfc → B, C, D
Annual rainfall (mm yr^−1^)	Continuous
Annual mean temperature ( °C)	Continuous

1 Texture classes according to WRB [41] and USDA [42].

2 Clay content class according to ÖNORM L 1050 [43].

## Data extraction and synthesis

Meta-data and relevant results for effect size calculation (mean, standard deviation and number of replicates) and moderator analysis were extracted from each study by the first author. When certain data was not provided, corresponding authors were contacted. Only one study per article or site was included to assure independence of effect sizes. When results were presented in form of figures, the software ImageJ V1.54.d was used to extract numbers. To calculate the variance and weight of each study, it is necessary to know the standard deviation. The EX-TRACT tool by Acutis et al. [32] allowed us to calculate standard deviations from the results of ANOVA and Multiple Comparison Test in cases where standard deviation or standard errors were not reported.

The effect size of each study (log response ratio) will be calculated according to Borenstein et al. [30]

(1) $$lnR=\ln\left(R\right)=\ln\left(\frac{\bar{x_{1}}}{{\bar{x}}_{2}}\right)=\ln\left(\bar{x_{1}}\right)-\ln\left({\bar{x}}_{2}\right)$$

where $x_{1}$ is the mean of treatment (CC) and ${\bar{x}}_{2}$ is the mean of control (no cover crop). Studies will be weighted by the inverse of variance

(2) $$w_{i}=\frac{1}{V_{i}+\tau^{2}}$$

where $V_{i}$ is the variance of the study *i* (within-study variance) and $\tau^{2}$ denotes the amount of residual heterogeneity (between-study variance). Effect size and the overall summary effect estimate will be calculated with the software MetaWin 2. We will perform random-effect meta-analysis, to account for both between- and within-study variance. A forest plot of the calculated effect sizes, including the summary effect size, will be provided for all SOC pools.

## Moderator and sensitivity analysis

Moderator effects on the pool specific SOC sequestration by cover crops will be analysed by sub-group analysis and meta-regression. Results will be presented in the form of figures and tables. Further, sensitivity analysis will be performed by assessing funnel plot asymmetry and using Egger's regression, which may indicate publication bias in meta-analysis; trim-and-fill analysis to allow one to enter values for “missing” studies; rank correlation analysis, to check the relationship between the effect size and variance and a fail-safe number to estimate how many missing studies we would need to retrieve and incorporate in the analysis before the p-value became non-significant [30]. These analyses will be done with MetaWin 2 and MetaWin 3.

## Data presentation and transparency

The complete database, including all extracted data as well as mean and standard deviation of control and treatment and calculated effect sizes will be made available in the data repository Zenodo. Moreover, a list of used literature will be provided. This way, we will make our work transparent and reusable.

## Ethics statements

The Authors followed MethodsX ethical guidelines, this work does not involve human subjects, animal experiments or data collected from social media.

## CRediT authorship contribution statement

**Julia Fohrafellner:** Methodology, Investigation, Data curation, Visualization, Writing – original draft. **Sophie Zechmeister-Boltenstern:** Methodology, Writing – review & editing, Supervision, Funding acquisition. **Rajasekaran Murugan:** Writing – review & editing, Supervision. **Katharina Keiblinger:** Writing – review & editing, Supervision. **Heide Spiegel:** Writing – review & editing. **Elena Valkama:** Conceptualization, Methodology, Supervision, Writing – review & editing, Project administration, Funding acquisition.

## Declaration of Competing Interest

The authors declare that they have no known competing financial interests or personal relationships that could have appeared to influence the work reported in this paper.

## Data Availability

No data was used for the research described in the article.

## Annex I

### Documentation of literature search

Search for scientific literature

Pre-screening in March 2022; WoS was searched, relevant literature (search string 1) was retrieved and checked for in- and exclusion. We re-defined our C and T and in- and exclusion criteria based on the literature available.

8.4.22

**Scopus:**

Search in: article title, abstract, keywords

Time frame: 1990 - present

Search string short 1 soil* AND (agr* OR farm*) AND (POM OR fPOM OR POC OR fPOC OR “particulate organic” OR MAOM OR MOM OR MAOC OR MinOM OR MASOC OR “mineral organic” OR “mineral-associated” OR oPOM OR “occluded POM” OR aggregate-occluded OR aggregate-associated OR microaggregate OR micro-aggregate) AND (“cover-crop*” OR “catch-crop*”)

106 articles exported to csv (excel) and into Jabref

**Web of Science:**

Searched in: topic

Time frame: 1990–01–01 – 2022–04–08 search string 1 soil* AND (agr* OR farm*) AND (diversif* OR (grass OR clover) ley* OR legume* OR intercrop* OR inter-crop* OR “intermediate crop*” OR cover-crop* OR catch-crop* OR “green manure” OR mixed-crop* OR undersown) AND (POM OR fPOM OR POC OR fPOC OR “particulate organic” OR MAOM OR MOM OR MAOC OR MinOM OR MASOC OR “mineral organic” OR “mineral-associated” OR oPOM OR “occluded POM” OR aggregate-occluded OR aggregate-associated OR microaggregate OR micro-aggregate) NOT (orchard OR forest OR fruit OR aquaculture OR aquiculture OR wood* OR vineyard OR arboricultu* OR horticult* OR rice OR olive OR *cane OR *tropic*)

113 results exported to excel and Jabref

**Science Direct:**

Searched in: Find articles with these terms

Time frame: 1990–2022

Search string short 4 (fitted for Science direct – only 8 operators and no wildcards) soil AND carbon AND (“particulate organic carbon” OR “mineral organic carbon” OR “mineral-associated carbon” OR “microbial carbon”) AND (“cover-crop” OR “catch-crop”)

187 results exported to (csv or excel not available) Jabref

**BASE:**

Searched in: Gesamtes Dokument

Time frame: 1990–2022

Search string short 5 (BASE) soil* AND (agr* OR farm*) AND (POM OR POC OR “particulate organic” OR MAOM OR MOM OR MAOC OR MinOM OR MASOC OR “mineral organic” OR “mineral-associated” OR oPOM OR “occluded POM” OR aggregate-occluded OR aggregate-associated OR microaggregate OR micro-aggregate) AND (“cover-crop*” OR “catch-crop*”)

78 results exported to Jabref (csv or excel not available)

11.04.22

**MDPI**

Searched in: logical operator, articles only

Time frame: until today (resulted in 1996–2022)

Search string short 6 (MDPI)

279 results

14.4.22

All results from databases in one subgroup in Jabref, total 536 – duplicated removed automatically and by choosing between entries/merging them. Also, non-English entries removed, posters, idea pitches on summer schools, book chapters, datasets. Master thesis and PhD thesis left for screening. Final: 322 entries

I exported the 322 articles into excel and started the screening with inclusion and exclusion criteria.

28.4.22

I noticed that microbial biomass keywords were absent when searching in:

**Scopus, WoS, Base**

I will apply adapted search strings, which include microbial biomass carbon, in these 3 databases and extract the articles which were missed in the first search on 8.4.22

**Scopus:**

Search in: article title, abstract, keywords

Time frame: 1990 - present

Search string short 1.2 soil* AND (agr* OR farm*) AND (POM OR fPOM OR POC OR fPOC OR “particulate organic” OR MAOM OR MOM OR MAOC OR MinOM OR MASOC OR “mineral organic” OR “mineral-associated” OR oPOM OR “occluded POM” OR aggregate-occluded OR aggregate-associated OR microaggregate OR micro-aggregate OR “microbial biomass carbon” OR MBC) AND (“cover-crop*” OR “catch-crop*”)

106 articles without MBC keywords (marked in yellow) – same number as on the 8.4.22. New search string including MBC keywords: 155 articles (meaning 49 new articles on MBC) - exported to csv (excel) and into Jabref

**Web of Science:**

Searched in: topic

Time frame: 1990–01–01 – 2022–04–08

Search string 1.2 soil* AND (agr* OR farm*) AND (diversif* OR (grass OR clover) ley* OR legume* OR intercrop* OR inter-crop* OR “intermediate crop*” OR cover-crop* OR catch-crop* OR “green manure” OR mixed-crop* OR undersown) AND (POM OR fPOM OR POC OR fPOC OR “particulate organic” OR MAOM OR MOM OR MAOC OR MinOM OR MASOC OR “mineral organic” OR “mineral-associated” OR oPOM OR “occluded POM” OR aggregate-occluded OR aggregate-associated OR microaggregate OR micro-aggregate OR “microbial biomass carbon” OR MBC) NOT (orchard OR forest OR fruit OR aquaculture OR aquiculture OR wood* OR vineyard OR arboricultu* OR horticult* OR rice OR olive OR *cane OR *tropic*)

113 articles without MBC keywords (marked in yellow) – same number as on the 8.4.22. New search string including MBC keywords: 202 articles - exported to csv (excel) and into Jabref

**BASE:**

Searched in: Gesamtes Dokument

Time frame: 1990–2022

Search string short 5.2 (BASE) soil* AND (agr* OR farm*) AND (POM OR “particulate organic” OR MAOM  OR MinOM OR MASOC OR “mineral organic” OR “mineral-associated” OR  “occluded POM” OR aggregate-occluded OR aggregate-associated  OR micro-aggregate OR “microbial biomass carbon” OR MBC) AND (“cover-crop*” OR “catch-crop*”)

→ I needed to delete some keywords to make room for the MBC keywords, as number of digits is limited

→ soil* AND (agr* OR farm*) AND (POM OR “particulate organic” OR MAOM OR MinOM OR MASOC OR “mineral organic” OR “mineral-associated” OR “occluded POM” OR aggregate-occluded OR aggregate-associated OR micro-aggregate OR “microbial biomass carbon” OR MBC) AND (“cover-crop*” OR “catch-crop*”)

**134** results exported to Jabref (csv or excel not available)

Articles previously extracted from MDPI and Science Direct were added. All results from databases in = 957 articles. Duplicates removed automatically and by choosing between entries/merging them. Also, non-English entries removed, posters, idea pitches on summer schools, some book chapters, datasets. Master and PhD thesis left for screening. Entries were exported into Excel and further duplicated and non-English articles were removed there.

Lastly, articles between the old and new article collections were fused/duplicates in new set removed.

**Start to search for grey literature**

7.7.22

**Google Scholar**

Search field only allows limited words (256 characters incl. spaces)

Searched in: normal search field

Time frame: no time frame, all

Sorted: According to relevance

Search string short 4 (fitted for Science direct – fits also for Google Scholar) soil AND carbon AND (“particulate organic carbon” OR “mineral organic carbon” OR “mineral-associated carbon” OR "microbial carbon") AND (“cover-crop” OR “catch-crop”)

7.7.22: 1310 results - cannot be exported, so I will screen them online.

8.7.22: 1310 results

I will start screening each article, regardless of scientific or not, and compare with scientific database to identify new literature. I will do so until no more relevant articles appear. This is possible as results are sorted according to relevance. All new articles, regardless of relevant or not, were added to the screening excel sheet and received an ID. All read articles on Google Scholar were marked/saved in “My library”.

In total, the first **100** entries were screened. **7** additional articles were obtained.

11.7.22

**BIORXIV**

Search field only allows limited words

Searched in: Search Terms & Keywords

Time frame: 1.1.1990 – 11.07.2022 (today)

Search string short 7 (BIORXIV) soil AND agricultur* AND carbon AND “cover crop”

1658 results

First 100 were downloaded

I will start screening each article and I will do so until no more relevant articles appear. This is possible as results are sorted according to relevance. All screened articles were added to the screening excel sheet and received an ID.

In total, the first 30 entries were screened. 0 additional articles were obtained.

**Final count of first search round**

Identified records:

Records identified from scientific databases: 957

From grey: 130

After duplicates removed:

Scientific: 507 (450 duplicates removed automatically)

Google scholar: 65 (65 duplicates removed by hand)

BIORXIV: 30 (0 duplicates removed)

Duplicates removed by JabRef software: 957–507= 450

Duplicates removed by hand: 130–65= 65

Total number of duplicates removed: 450+65= 515

Scientific and grey literature that was screened: *n* = 602

Records from reviews: 3

Scientific and grey literature + literature from reviews that was screened: *n* = 605

**Second search round**

1.6.2023

Search for suitable literature in **EJP SOIL LTE Database**

Cite as: “EJP7.3 Database contributors, 2021. EJP SOIL Long-term field experiment”

2 articles found

D'Hose 2015 - MBC

Lammerding 2015 – MBC

June 2023

Hu et al. 2023 MAOC and MBC screened for relevant literature – no new articles found

Qijuan Hu, Ben W. Thomas, David Powlson, Yingxiao Hu, Yu Zhang, Xie Jun, Xiaojun Shi, Yuting Zhang, Soil organic carbon fractions in response to soil, environmental and agronomic factors under cover cropping systems: A global meta-analysis, Agriculture, Ecosystems & Environment, Volume 355, 2023, 108,591, ISSN 0167–8809, ht tps://doi.org/10.1016/j.agee.2023.108591.

19.06.23

**Science Direct:**

Searched in: Find articles with these terms

Time frame: 2022–2023

Search string short 4 (fitted for Science direct – only 8 operators and no wildcards) soil AND carbon AND (“particulate organic carbon” OR “mineral organic carbon” OR “mineral-associated carbon” OR "microbial carbon") AND (“cover-crop” OR “catch-crop”)

65 articles imported into jabref

**Scopus:**

Search in: article title, abstract, keywords

Time frame: 2022 - present

Search string short 1.2 soil* AND (agr* OR farm*) AND (POM OR fPOM OR POC OR fPOC OR “particulate organic” OR MAOM OR MOM OR MAOC OR MinOM OR MASOC OR “mineral organic” OR “mineral-associated” OR oPOM OR “occluded POM” OR aggregate-occluded OR aggregate-associated OR microaggregate OR micro-aggregate OR “microbial biomass carbon” OR MBC) AND (“cover-crop*” OR “catch-crop*”)

33 articles imported into jabref

**Web of Science:**

Searched in: topic

Time frame: 2022–04–08 – 2023–06–19

Search string 1.2 soil* AND (agr* OR farm*) AND (diversif* OR (grass OR clover) ley* OR legume* OR intercrop* OR inter-crop* OR “intermediate crop*” OR cover-crop* OR catch-crop* OR “green manure” OR mixed-crop* OR undersown) AND (POM OR fPOM OR POC OR fPOC OR “particulate organic” OR MAOM OR MOM OR MAOC OR MinOM OR MASOC OR “mineral organic” OR “mineral-associated” OR oPOM OR “occluded POM” OR aggregate-occluded OR aggregate-associated OR microaggregate OR micro-aggregate OR “microbial biomass carbon” OR MBC) NOT (orchard OR forest OR fruit OR aquaculture OR aquiculture OR wood* OR vineyard OR arboricultu* OR horticult* OR rice OR olive OR *cane OR *tropic*)

26 articles imported into jabref

124 articles – 17 duplicates removed; 1 article removed; total 106 new articles screened for relevance (title and abstract, then full article)

**Specific searches**

22.06.23

Search for articles with C in different aggregate sizes

**Science Direct:**

Searched in: Find articles with these terms

Time frame: 1990–2023

Search string short 4.2aggregates  (fitted for Science direct – only 8 operators and no wildcards) soil AND carbon AND AND (“carbon in aggregate” OR “carbon fraction”) AND (“cover-crop” OR “catch-crop”)

214 results imported into Jabref

**Scopus:**

Search in: article title, abstract, keywords

Time frame: 1990 - present

Search string short 1.2.2aggregates soil* AND (agr* OR farm*) AND (“carbon in aggregate*” OR “carbon fraction*”) AND (“cover-crop*” OR “catch-crop*”)

17 results imported into Jabref

**Web of Science:**

Searched in: topic

Time frame: 1990–01–01 – 2023–06–19

Search string 1.2.2aggregates soil* AND (agr* OR farm*) AND (diversif* OR (grass OR clover) ley* OR legume* OR intercrop* OR inter-crop* OR “intermediate crop*” OR cover-crop* OR catch-crop* OR “green manure” OR mixed-crop* OR undersown) AND (“carbon in aggregate*” OR “carbon fraction*”) NOT (orchard OR forest OR fruit OR aquaculture OR aquiculture OR wood* OR vineyard OR vegetable* OR arboricultu* OR horticult* OR olive OR *cane OR *tropic*)

42 results imported into Jabref

273 results – duplicates removed – duplicates with first and second search results removed – book chapters and empty cells removed – 117 duplicates removed - 156 results screened for relevance

23.06.23

Search for articles with upland rice

**Science Direct:**

Searched in: Find articles with these terms

Time frame: 1990–2023

Search string short 4.3.rice (fitted for Science direct – only 8 operators and no wildcards) soil AND carbon AND rice AND (“particulate organic carbon” OR “mineral-associated carbon” OR “microbial biomass carbon”) AND (“cover-crop” OR “catch-crop”)

191 results

And soil AND carbon AND rice AND (“particulate organic matter” OR “mineral-associated matter” OR “microbial biomass matter”) AND (“cover crop” OR “catch crop”)

98 results all imported into Jabref

**Scopus:**

Search in: article title, abstract, keywords

Time frame: 1990 - present

Search string short 1.2.2.rice soil* AND rice AND (POM OR fPOM OR POC OR fPOC OR “particulate organic” OR MAOM OR MOM OR MAOC OR MinOM OR MASOC OR “mineral organic” OR “mineral-associated” OR oPOM OR “occluded POM” OR aggregate-occluded OR aggregate-associated OR microaggregate OR micro-aggregate OR “carbon in aggregate*” OR “fraction*”) AND (“cover crop*” OR “catch crop*”)

10 results imported into Jabref

**Web of Science:**

Searched in: topic

Time frame: 1990–01–01 – 2023–06–19

Search string 1.2.2.rice soil* AND rice AND (diversif* OR (grass OR clover) ley* OR legume* OR intercrop* OR inter-crop* OR “intermediate crop*” OR cover-crop* OR catch-crop* OR “green manure” OR mixed-crop* OR undersown) AND (“carbon in aggregate*” OR fraction*) NOT (orchard OR forest OR fruit OR aquaculture OR aquiculture OR wood* OR vineyard OR vegetable* OR arboricultu* OR horticult* OR olive OR *cane)

132 results imported into Jabref

SUM: 240 → duplicates removed be comparing with results from previous screenings → 222 results into excel for screening
